# Bias in a protocol for a meta-analysis of 5-HTTLPR, stress, and depression

**DOI:** 10.1186/1471-244X-14-179

**Published:** 2014-06-17

**Authors:** Terrie E Moffitt, Avshalom Caspi

**Affiliations:** 1 Department of Psychology and Neuroscience, Duke University, Suite 201 Grey House, 2020 West Main St., Box 104410, Durham NC 27708, USA; 2 Institute of Psychiatry, King’s College London, London, UK

## Bias in a protocol for a meta-analysis of 5-HTTLPR, stress, and depression

We are delighted to see pre-study publication in *BMC Psychiatry*[1] of the research design and plans for meta-analysis of the literature on the interaction between serotonin transporter genotype and life stress, predicting depression [2]. However, we recommend two changes: dropping “Primary Analysis Plan 2” and including studies in the meta-analysis with sample sizes under 300 participants. In mid-2012 we wrote to the group of researchers involved in the meta-analysis, expressing these two concerns with the plan. Because these concerns have not been adequately addressed in the published protocol, we submit this correspondence.

### Issue 1: “Primary Analysis Plan 2” to study lifetime depression does not allow for establishing temporal order between stress and depression

Primary Analysis Plan 2 in Culverhouse et al. [1] states that studies that measured stress and depression using lifetime measurement will be included in the meta-analysis. However, these studies should not be used in the forthcoming meta-analysis because using lifetime measures precludes establishing temporal order between a hypothesized cause and a hypothesized effect. The hypothesis in question is that individuals with an at-risk serotonin transporter genotype are likely to develop depression after life stress and in response to it. The minimal criterion for a valid test of this hypothesis is a set of measures that can unambiguously establish that the stress came before the depression. When using lifetime measures one cannot simply make the assumption that stress came before the depression, because there is a literature showing that individuals with depression tend to experience more stressful life events as a consequence of their mood disorder [3]. This well-known phenomenon is referred to in the literature as “stress generation” [4]. For example, depressed individuals have elevated rates of intimate partner violence and divorce. To use retrospective reports of lifetime depression in a test of this GxE hypothesis is tantamount to using lifetime weight to test hypotheses about the cause of low birth-weight, or to use lifetime IQ to test hypotheses about causes of IQ decline in Alzheimer’s dementia; the measure sounds the same, but it is not. Timing is everything. The importance of temporal order in hypothesis testing in studies having observational designs is nicely explained in a powerpoint lecture "What Do Survey Data Really Mean? Considering Issues of Causality and Temporality in Survey Research," by Seth Noar (http://www.nidcr.nih.gov/Research/DER/BSSRB/PowerPointPresentations/default.htm). Strong GxE tests documenting that stress antedated depression exist, but are not included in the meta-analysis (e.g., [5]).

Setting aside for the moment the question of temporal order between cause and effect, studies using lifetime measures should not be used because these measures are inaccurate, inadequate, and misleading as measures of depression and stress. The literature contains ample documentation that retrospective recall of lifetime depression is inadequate for research purposes. We reviewed this evidence in *Psychological Medicine*[6], and since then the inadequacy of retrospective recall of lifetime depression has been demonstrated in multiple studies [7]–[9]. Together these and prior papers show that retrospective lifetime reports overlook at least half of depression cases. Thus Plan 2 of the meta-analysis will wrongly assign many individuals who have had depression to the non-depressed outcome group. Numerous publications have noted that retrospective checklists of lifetime stressful life events are likewise unsuitable for research purposes [10,11]. In relation to the subject of the proposed meta-analysis, GxE research, the poor accuracy of these retrospective recall measures is particularly important. Simulation studies reveal that the difference between measurements that are unreliable (correlation with true score = 0.4) vs reliable (0.7) corresponds to a large difference in sample size. Thus, although measuring environmental exposure is costly, doing it well can pay for itself by reducing sample size [12]. However, our concern is that lifetime measures of stress and depression in the forthcoming meta-analysis are not merely unreliable, they are also invalid, and therefore they contaminate the meta-analysis with misinformation. Increased power afforded by larger N sometimes counterveils unreliable measurement, but large N cannot counterveil invalid data. Unfortunately, the biasing influence of invalid data in a meta-analysis is exacerbated by large samples.

The *BMC Psychiatry* methods paper includes two plans. It includes Primary Analysis 1, a new separate plan to study only those data sets that can establish temporal order between stress and depression. We applaud the addition of Plan 1. The paper also retains Primary Analysis 2, the original plan to study lifetime depression including all studies available, which we argued above is inappropriate. We must query why Plan 2 is still retained. The abovementioned flaws in Plan 2 should come as no surprise, because our point about the importance of accurate measurement of temporal order is not new. We addressed it empirically in our original *Science* paper reporting the GxE in question [2]. In that paper we estimated the GxE effect using a measure of life events that occurred prior to depression and we estimated it again using a measure of life events that occurred after depression. Results showed empirically that unless the stress occurred before the depression, the GxE finding was not observed. Culverhouse et al. carefully and rightly emphasize the importance of matching the design features of a replication analysis as closely as possible to design features of the original publication. However, Plan 2 not only fails to match the design of the original publication, it includes a design feature that the original publication tested and advised against.

We suspect that Plan 2 is retained solely because it offers an attention-getting large sample size. To quote Culverhouse et al. [1], “Our second set of primary analyses will involve larger sample sizes, including children and adults of all ages. The increase in sample size will result in increased power if there is a broad genetic association between 5-HTTLPR genotypes, stress, and depression. However, this comes at a cost; in these analyses, we give up the opportunity to investigate whether stress preceding depression was a potential cause of the depression, as relative timing of stress and depression may not be known, and thus will not be included in the models.” We anticipate that even if the more focused Analysis 1 (closer design replication, smaller N) shows evidence of the interaction, the results of Plan 2 (which is not a replication, but has a larger N) will be those most likely to be highlighted by the authors, covered in the media, and remembered by the public. Plan 2’s result will be rendered more salient to readers because of its anticipated sample size exceeding 30,000 participants. This appears to be the rationale for retaining this plan, despite the fact that its design was shown to be flawed by Caspi et al. [2]).

### Issue 2: The protocol excludes studies with N < 300

The protocol excludes many important studies, in part because of their design features (e.g., case-only designs; [13]) or because they reported symptom dimensions rather than categorical diagnoses of depression (e.g., [14]). However, here we focus on sample size as this has been pivotal in the debate. Discovery science in genetics requires large samples, but hypothesis-testing science does not necessarily. The Culverhouse et al. replication project is not discovery science, it is hypothesis-testing science. In hypothesis-testing science, the consideration of sample size is secondary to more primary considerations of quality of the measures and correctness of design. This order of priorities may be particularly true of hypothesis testing using a meta-analysis approach, as the approach itself provides more than ample sample size. Many of the best-designed studies for testing the GxE hypothesis in question have samples under 300; these smaller studies are significantly more likely to be prospective-longitudinal and to utilize face-to-face interviews [15]. These smaller studies are also more likely to be able to establish temporal order between cause (stress) and effect (depression). In particular, studies of medical illness stressors overcome the problems of variable stressors between subjects and inaccurate retrospective assessment that compromise power in many other GxE studies. However these medical-stressor studies are typically small, and as a result the protocol plan has excluded them. Some studies the protocol includes, no matter how large, must be designated unsuitable for this project if their measures of stress and depression are weak on validity, as is common when data must be collected through the post, telephone, or internet to contain costs of assessing a large sample. When it comes to measuring stress and depression, face-to-face clinical interviews have superior reliability and validity but are more expensive, usually necessitating smaller samples. Again, Culverhouse et al. have emphasized the importance of matching features of a replication analysis as closely as possible to features of the original published study. The original published study used face-to-face clinical interviews. Thus, the protocol plans to include studies that fail to match the design of the original publication in the key area of measurement, and most such studies have very large Ns. Moreover, as noted above, large-N studies are even more unsuitable if their designs do not allow establishing clear temporal order between hypothetical cause and outcome. The protocol’s over-emphasis on sample size of individual studies, coupled with exclusion of many well-designed studies for testing the hypothesis, is misguided.

The rationale given in the Culverhouse et al. protocol for exclusion of small studies is that more small studies have claimed positive findings. They note small-N studies run a risk of publication bias. Such bias emerges when a small-N study with a negative finding is more often “file-drawered” because it is not deemed rigorous enough to constitute decisive rejection of the null, whereas a small-N study with a positive finding would be more often published because it was able to reject the null despite being under-powered. However, the simple fact that more small studies have obtained positive findings does not by itself constitute evidence of such publication bias, particularly when there are systematic differences in quality between small studies and large studies. Moreover, it has been commented before that in relation to this particular GxE finding, both researchers and editors have been quite keen to publish negative findings (6 nagative reports have appeared in the last 3 years, all of which are taking part in the meta-analysis, although curiously most positive reports appearing over this time period have not been invited to take part). Culverhouse et al. allow unpublished studies to submit data for the meta-analysis, and they report that they have trawled for these unpublished studies. As such, requiring N > 300 to prevent the file drawer problem does not seem necessary.

Our point about sample size is not new. We explained it in our *American Journal of Psychiatry* paper [16], Uher et al. explained it in two publications [15,17], and Karg et al. also explained it in their meta-analysis [18]. Yet, the meta-analysis protocol does not contain a justification of its choice of N = 300 as a cut off for study inclusion. Why not 500, why not 200? According to PRISMA guidelines for reporting meta-analyses, those that aspire to be authoritative provide a rationale for their decision points, e.g., “Specify study characteristics used as criteria for eligibility, giving rationale” (http://www.prisma-statement.org/2.1.2%20-%20PRISMA%202009%20Checklist.pdf).

Culverhouse et al. include an a priori plan to test for effects of study design features on heterogeneity in findings, and include a list of five design features to be tested. We applaud this approach. However, the list of design features to be tested omits sample size. We find this omission curious because sample size has been at the heart of debate in the literature about prior meta-anlayses of this GxE. The heterogeneity analyses proposed by Culverhouse (cross-sectional vs. longitudinal, interview vs. questionnaire, specific stressor vs. undifferentiated stressor) are important analyses to guide the field going forward. Unfortunately, because so many high-quality longitudinal, interview-based, and specific-stressor studies have been excluded by the sample-size restriction, the results of the planned analyses will be difficult to interpret. Excluding small studies instead of testing for their putative bias on findings seems a missed opportunity for the Culverhouse team. In fact, our claim is not really that smaller studies are more desirable. Our claim is that the largest studies are least desirable because they have the worst measurement technology and in many cases have been unable to establish temporal order, which is rather different. Including a test of sample size as a heterogeneity factor could shed light on the veracity of our claim.

These two issues that we raise here, temporal order and sample size, are not new to observational hypothesis-testing research. They apply to all observational studies, beyond the special case of GxE studies. Other meta-analyses of this GxE hypothesis have made these same methodological mistakes before, and these mistakes have been repeatedly pointed out in published articles in the past five years. As such, the protocol as published seems fundamentally and inexplicably flawed. As we said in our 2012 letter to the meta-analysis collaborators, we regret this missed opportunity to do something better.

## Competing interests

Through the Wisconsin Alumni Research Foundation Drs. Moffitt and Caspi hold patent “Method for Assessing a Behavioral Disposition” (U.S. Patent Office serial number 10/889,450). No licenses are issued.

## Authors’ contributions

TM and AC wrote the letter and gave final approval of the version to be published.

## Weblinks

http://www.moffittcaspi.com/sites/moffittcaspi.com/files/Letter_to_Culverhouse_JUNE2012.pdf

http://www.nidcr.nih.gov/Research/DER/BSSRB/PowerPointPresentations/default.htm

http://www.prisma-statement.org/2.1.2%20-%20PRISMA%202009%20Checklist.pdf

## Pre-publication history

The pre-publication history for this paper can be accessed here:

http://www.biomedcentral.com/1471-244X/14/179/prepub
